# Dihydroarteannuin Ameliorates Collagen-Induced Arthritis *Via* Inhibiting B Cell Activation by Activating the FcγRIIb/Lyn/SHP-1 Pathway

**DOI:** 10.3389/fphar.2022.883835

**Published:** 2022-05-03

**Authors:** Congqi Hu, Danbin Wu, Jiahui Yu, Jia Xu, Lijuan Liu, Mingying Zhang, Wei Jiao, Guangxing Chen

**Affiliations:** ^1^ First Clinical Medical School, Guangzhou University of Chinese Medicine, Guangzhou, China; ^2^ Department of Rheumatology, The First Affiliated Hospital of Guangzhou University of Chinese Medicine, Guangzhou, China; ^3^ Lingnan Medical Research Center of Guangzhou University of Chinese Medicine, Guangzhou, China; ^4^ Baiyun Hospital of The First Affiliated Hospital of Guangzhou University of Chinese Medicine, Guangzhou, China

**Keywords:** dihydroarteannuin, FcγRIIb, rheumatoid arthritis, ST486, CIA mice

## Abstract

**Background:** Dihydroarteannuin (DHA), which is extracted from the traditional Chinese herb *Artemisia annua L*, exhibits potent immunosuppressive activity in rheumatoid arthritis (RA). Strong evidence indicates that B cells act as an essential factor in the pathogenesis of RA, but research on the immunosuppressive function of DHA in regulating B cells is limited.

**Objective:** To investigate the modulatory effects of DHA on joint destruction, proinflammatory cytokine production, activation, apoptosis and proliferation of B cells and to explore the possible associated mechanism in RA treatment.

**Methods:** Collagen-induced arthritis (CIA) model was established. Weight and joint oedema were record weekly, and joint damage was detected by micro-CT scan. Human Burkitt B lymphoma cells lacking endogenous Fc gamma receptor b (FcγRIIb) gene were transfected with a 232Thr loss-of-function mutant to construct a mutant cell model ST486. The proliferation of ST486 cells was assessed with Cell Counting Kit-8. Apoptosis and activation were tested by flow cytometry. The effects of DHA on the activation of FcγRIIb, protein tyrosine kinases (Lyn), and SH2-containing tyrosine phosphatase-1 (SHP-1) signaling pathways were determined by western blotting.

**Results:** In comparison to model group, bone volume/tissue volume (BV/TV) and bone mineral density (BMD) were increased, whereas joint oedema was decreased in both of the DHA and MTX group. The mRNA and protein expression levels of Interleukin-6 (IL-6) and Tumor necrosis factor-alpha (TNF-α) were decreased after treatment with DHA. In addition, DHA treatment promoted the apoptosis, inhibited the activation and proliferation of ST486 cells. Furthermore, the protein expression levels of FcγRIIb, SHP-1, and Lyn were increased after treatment with DHA. Moreover, the expression of phosphorylated CD19 was also inhibited by DHA.

**Conclusion:** We provide the first evidence that DHA may alleviate collagen-induced arthritis by activating the FcγRIIb/Lyn/SHP-1 signaling pathway in B cell, indicating that DHA is a novel and valuable candidate for RA therapy.

## Highlights

• Studies on the immunosuppressive function of DHA in regulating B cells are limited.
• Restoring the inhibitory function of FcγRIIB may be a new strategy for the treatment of RA.
• We used gene transfection technique to construct FcγRIIB mutant cell line ST486.
• DHA inhibits B cell activation by activating the FcγRIIb/Lyn/SHP-1 pathway.
• This is the first report showing that DHA achieves these effects by activating this pathway.

## Introduction

Rheumatoid arthritis (RA) is a common autoimmune disease, which seriously endangers human health. Although the etiology is still unknown, many studies have shown that genetic defects and environmental factors interact to cause the pathogenesis of RA (Smolen et al., 2016). Genetic, immunological and clinical studies show that B cells play a key role in the occurrence, development and treatment of RA. Activated B cells produce a large number of antibodies against autoantigens, which is one of the main differences between RA and other inflammatory arthritis, including rheumatoid factors (RF) and anti-cyclic citrullinated peptide (anti-CCP) antibodies, which are present in patients’ sera before the clinical symptoms of RA (van Gaalen et al., 2005). Additionally, a randomized clinical study have shown that Rituximab, a monoclonal antibody against B cell, not only significantly improves the autoantibody level of RA patients, but also reduces radiological progress. Its efficacy is closely related to the clearance of memory B cells, strongly suggesting the important role of B cells in RA (Nakou et al., 2009; Chatzidionysiou et al., 2016).

Fc gamma receptor b (FcγRIIb) is an important molecule in immune regulation, which can prevent excessive activation of B cell receptor (BCR) signals and reduce the risk of autoimmune diseases. The down-regulation or functional deficiency of FcγRIIb will not only increase the incidence of RA or systemic lupus erythematosus (SLE), but also aggravate the joint destruction of RA (Nimmerjahn and Ravetch, 2006). In addition, a study of single nucleotide polymorphism (SNP) in 246 patients with RA was followed up for 6 years and it was found that the 695T > C (Ile232Thr) polymorphism in exon five of FcγRIIb gene could attenuate the signal of inhibitory receptor and aggravate the condition of RA and joint destruction (Li et al., 2003; Radstake et al., 2006). More importantly, a clinical study showing that FcγRIIb can be regulated, it is dysfunctional in active RA but normal in inactive RA where the disease is controlled (Magnusson et al., 2014). Additionally, after FcγRIIb binds to the BCR and tyrosine phosphorylates, it can bind to SH2-containing tyrosine phosphatase-1(SHP-1). The effect of SHP-1 on the function of B cells is not only on their proliferative capacity, but also on apoptosis and cell killing, which can be regulated by interacting with different molecules downstream of B cell surface receptors (Raghav et al., 2018a; Raghav et al., 2018b; Raghav et al., 2018c).

Disease-modifying antirheumatic drugs (DMARDs) are the basic drugs for the treatment of RA (Singh et al., 2015), especially methotrexate (MTX) is the anchoring drug of RA. In spite of this, these drugs still have many side effects (Otero et al., 2017). Therefore, the development of alternative drugs with fewer side effects is very important to achieve a better clinical outcome (Zhang et al., 2010).

Dihydroarteannuin (DHA; MW: 284.35, molecular formula: C_15_H_24_O_5_), a semisynthetic derivative of artemisinin, is a widely used antimalarial drug with anti-inflammatory, anticancer, and immunosuppressive activities (Yu et al., 2021). Increasing evidence suggests that DHA exerts an immunosuppressive function in several autoimmune diseases, including RA and SLE (Li et al., 2006; Fan et al., 2018a; Li et al., 2019; Wang et al., 2021). Previous studies have found that DHA promotes apoptosis in B cells and that B cell lymphoma-2 (Bcl-2) is an anti-apoptotic protein that determines B cell apoptosis by interacting with pro-apoptotic members of the Bcl-2 family (Raghav et al., 2012a; Raghav et al., 2012b; Raghav et al., 2019). Therefore, we speculate that DHA may promote B-cell apoptosis by inhibiting Bcl-2 to achieve therapeutic effects in RA. In addition, some studies have demonstrated that DHA can diminish CD8+T cell memory, affect Th and regulatory T cell functions, restore the Treg/Th17 cell balance, and so on (Zhao et al., 2012; Fan et al., 2018b; Chen et al., 2020). However, how DHA affects the function of B cells is still unclear. More importantly, the potential immune regulation mechanism of DHA is worthy of further study.

In the present study, CIA in DBA/1 mice was established. Human Burkitt B lymphoma cells (lacking endogenous FcγRIIb gene) were transfected with a 232Thr loss-of-function mutant to construct a mutant cell model ST486. We investigated the effects of DHA on joint damage in CIA mice, and the proinflammatory cytokine production, activation, proliferation and apoptosis of ST486 cells. In addition, we further confirmed whether these effects correlated with the FcγRIIb/Lyn/SHP-1 signaling pathway.

## Materials and Methods

### Chemicals and Reagents

DHA (D7439, 150 mg), lipopolysaccharide (LPS), phorbol myristate acetate (PMA) and MTX hydrate (M8407) were purchased from Sigma-Aldrich (St. Louis, MO, United States). Complete Freund’s adjuvant (CFA) (7001, 10 ml), incomplete Freund’s adjuvant (IFA) (7002, 10 ml), and bovine type II collagen (20021, 10 ml) were purchased from Chondrex, Inc. (Redmond, WA, United States). ST486 cell line (70014502, CRL-1647), RPMI 1640 culture medium and fetal bovine serum (FBS) were purchased from ATCC Co., Ltd. (United States). AffiniPure goat anti-human IgM (109-005-129) was obtained from Jackson ImmunoResearch Co., Ltd. (PA, United States). Fluo-4 AM (F312) was purchased from Dojindo Co., Ltd. (Japan). The primary antibody against CD32B (ab45143, EP888Y) was purchased from Abcam Co., Ltd. (Cambridge, United Kingdom). The primary antibody against GAPDH (10494-1-AP) was obtained from Proteintech Group, Inc. (United States). The primary antibodies against Lyn (C13F9) (2796), SHP-1 (C14H6) (3759), and phospho-CD19 (Tyr531) (3571) were purchased from CST Co., Ltd. (United States).

### Animals

The animals used for this experiment were male DBA/1J mice (Vital River Laboratory Animal Technologies Co. Ltd. Beijing, China) that were 8 weeks old. The mice were housed in a laminar flow cabinet with a 12 h light/dark cycle and maintained on specific pathogen-free (SPF) laboratory chow and water ad libitum. All the experimental studies were strictly in accordance with Guangzhou University of Chinese Medicine Animal Ethics Committee guidelines for the rational use of animals.

### Induction and Evaluation of Collagen-Induced Arthritis

After 1 week of adaptation, 24 DBA/1J mice were randomly divided into two groups: control group (*n* = 6), arthritis-induced group (*n* = 18). To prepare mouse CIA model, 0.1 ml of emulsion of bovine type II collagen and CFA (1:1, v/v) was injected intradermally into the tail base of mice in arthritis-induced group on day 0, as the primary immunization. A booster injection of 0.1 ml of emulsion of bovine type II collagen and IFA (1:1, v/v) was administered intradermally into the back on day 21, as the secondary immunization (Zhang et al., 2013; Miyoshi and Liu, 2018). Mice in control group injected normal saline at the same location and frequency as arthritis-induced group. The clinical symptoms were recorded weekly since the secondary immunization, with the arthritis index scores according to the following criteria: 0 = normal; 1 = erythema and mild swelling; 2 = erythema and swelling extending to ankle joints and one or two toes; 3 = erythema and swelling extending to metatarsal joints and more than two toes; and 4 = ankylosing deformity with joint swelling. The scores from each paw were added to obtain a cumulative score between 0 and 16. In addition, swelling in the paw was measured using digital calipers, and body weight was recorded during the course.

### Drug Administration

After the secondary immunization on day 21, mice in the arthritis-induced group were randomly divided into three groups (*n* = 6 per group): model group, DHA group (20 mg/kg, daily), MTX group (2 mg/kg, every 3 days). DHA and MTX was dissolved in corn oil and administrated by oral gavage from day 21 to day 49. Control and model mice were orally given an equal volume of corn oil in parallel.

### Micro-CT Analysis

After 4 weeks of drug administration, the right hind knee and ankle of each mouse was withdrawn and scanned by Skyscan 1176 micro-CT scanner (Bruker micro-CT, Kontich, Belgium). The scanning was carried out using following settings: voltage, 80 kV; source current, 88 μA; pixel size 4 μm. Two and three-dimensional images were generated using Data-viewer and CTvol softwares (Bruker micro-CT, Kontich, Belgium) respectively. The bone mineral density (BMD) and bone volume/tissue volume (BV/TV, %) were measured using CT Analyser program (Bruker micro-CT, Kontich, Belgium).

### Plasmid Constructs, Transfections, and ST486 Cell Culture

ST486 cells were maintained in RPMI-1640 medium in a humidified chamber with 37°C at 5% CO_2_. ST486 cells that expressed wild-type human FcγRIIb or the I232T loss-of-function mutant were constructed. Murine stem cell virus (pMSCV) puro plasmids contained human FcγRIIb-I232T or FcγRIIb-WT were used as templates, and inserted into lentiviral expression vector (pLVX) *via* the EcoRI and MluI restriction enzyme sites after PCR amplification. Then the expression vector constructs of human FcγRIIb-WT or FcγRIIb-I232T were transfected to ST486 by electroporation. The stable sublines of ST486 cells expressing equivalent levels of human FcγRIIb-I232T or FcγRIIb-WT were sorted by at least two rounds of fluorescence-activated cell sorting. Quantitative real-time PCR (qRT-PCR) assays were performed to verify the transfection. The methods for RNA purification and qRT-PCR will be described below. The specific primer sets in Supplementary Table S1. After the transfection, transfected cells (Mut group) were incubated with or without different concentrations of DHA or MTX to perform the following studies.

### Measurement of Proinflammatory Cytokine Levels by Enzyme-Linked Immuno Sorbent Assay

ELISA kit (Beijing 4A Biotech Co., Ltd., Beijing, China) were used to determine the cytokine production under DHA treatment. Transfected cells (Mut group) were divided into six groups: NC (without DHA and LPS), LPS (150 ng/ml), DHA (250 ng/ml), DHA (500 ng/ml), DHA (1000 ng/ml), and MTX (500 ng/ml, positive control group). Twenty-4 hours after transfection, the cells (1 × 10^5^/ml) were seeded in 24-well plates and treated with different concentrations of DHA or MTX (500 ng/ml) for 48 h, then PMA was added and incubated at 37°C with 5% CO_2_ for 48 h. The supernatant was then discarded, LPS (150 ng/ml) was added to each group except the NC group, and the cells were incubated overnight at 37°C with 5% CO_2_. The culture supernatants were collected, and the amount of TNF-α released from the cells was detected according to the manufacturer’s instructions. IL-6 cytokine assays were carried out using the same method.

### RNA Purification and a Quantitative Real-Time PCR Assay

qRT-PCR was performed to analyze the expression of TNF-α and IL-6 in ST486 cells treated with DHA or MTX. Total RNA was isolated with TRIzol (Invitrogen, United States) and reverse transcribed into cDNA using a Prime Script RT Reagent kit (Takara Biotechnology, Dalian, China) according to the manufacturer’s protocol. qRT-PCR was completed after 40 cycles of 95°C for 30 s, 60°C for 1 min, and 72°C for 1 min. The relative fold-changes of gene transcriptions were determined by 2^−△△Ct^ method with the specific primer sets in Supplementary Table S1.

### Cell Viability Assay

The experiment was divided into six groups, which involved FcγRIIb-WT (control group), FcγRIIb-Mut (model group), MTX (500 ng/ml, positive control group), DHA (250 ng/ml), DHA (500 ng/ml) and DHA (1000 ng/ml). 10 μL of CCK-8 solution was added after culturing with different concentrations of DHA or MTX for 48 h. Then cells were incubated under the same conditions for another 2 h. The absorbance at 490 nm was measured by a microplate reader (iMark, Thermo Fisher Scientific, United States) to evaluate cell viability.

### Apoptosis Assays

Annexin V apoptosis detection kit (BD Biosciences, United States) was used to measure cell apoptosis. After treating with different concentration of DHA or MTX as described above, the transfected cells were harvested and incubated with 7-AAD and Annexin V antibody following the manufacturer’s instructions. Then, cells were immediately analyzed by a FACSCalibur flow cytometer.

### Measurement of Cytoplasmic Ca^2+^ Concentrations

Multiple signal transduction pathways can affect changes in intracellular Ca^2+^ concentration, and thus the detection of intracellular Ca^2+^ changes can help to understand the initiation, enhancement or inhibition of cellular functions. In this study, we used flow cytometry to monitor the dynamic changes of intracellular Ca^2+^ concentration in order to dynamically observe the degree of B-cell activation and thus verify whether DHA can inhibit the B-cell activation. After treating with different concentration of DHA or MTX as described above, the transfected cells (1.5 × 10^6^/ml) were incubated in RPMI 1640 medium containing Fluo-4 AM (5 μΜ) for 1 h in a humidified chamber with 37°C at 5% CO_2_ to load with Fluo-4 AM. Then it was washed 3 times and detected by a flow cytometer constantly. The cells were stimulated with anti-human IgM (20 μg/ml) at 50 s, CaCl_2_ was added back to a final concentration of 2 mM at 400 s, and intracellular Ca^2+^ flux was measured for 800 s.

### Western Blot Analysis

After treating with different concentration of DHA or MTX as described above, cells were stimulated with anti-human IgM (20 μg/ml) for 15 min. Protein lysates obtained from equal numbers of ST486 cells were separated by SDS-PAGE and then transferred to 0.4 μm PVDF membranes *via* electroblotting. After being blocked by skimmed milk for 1 h, membranes were incubated with antibodies against GAPDH, phospho-CD19, CD32B, Lyn, and SHP-1 overnight at 4°C. Then the membranes were incubated with the corresponding secondary antibodies at room temperature for 1 h. GAPDH was used as an internal control. Immunoreactive bands were visualized through Enhanced Chemiluminescence method and recorded in gel documentation system.

### Statistical Analysis

All experiments were repeated at least three times, and all data are presented as the mean ± SEM. Statistics were analyzed using SPSS (version 20.0, IBM, Armonk, NY) while graphs were drawn by GraphPad Prism software (version 7.0). Significant differences between multiple groups were calculated using one-way ANOVA followed by Tukey’s test. Values of *p* < 0.05 were considered significant.

## Results

### Dihydroarteannuin Alleviated Joint Destruction in Collagen-Induced Arthritis Mice

**FIGURE 1:**
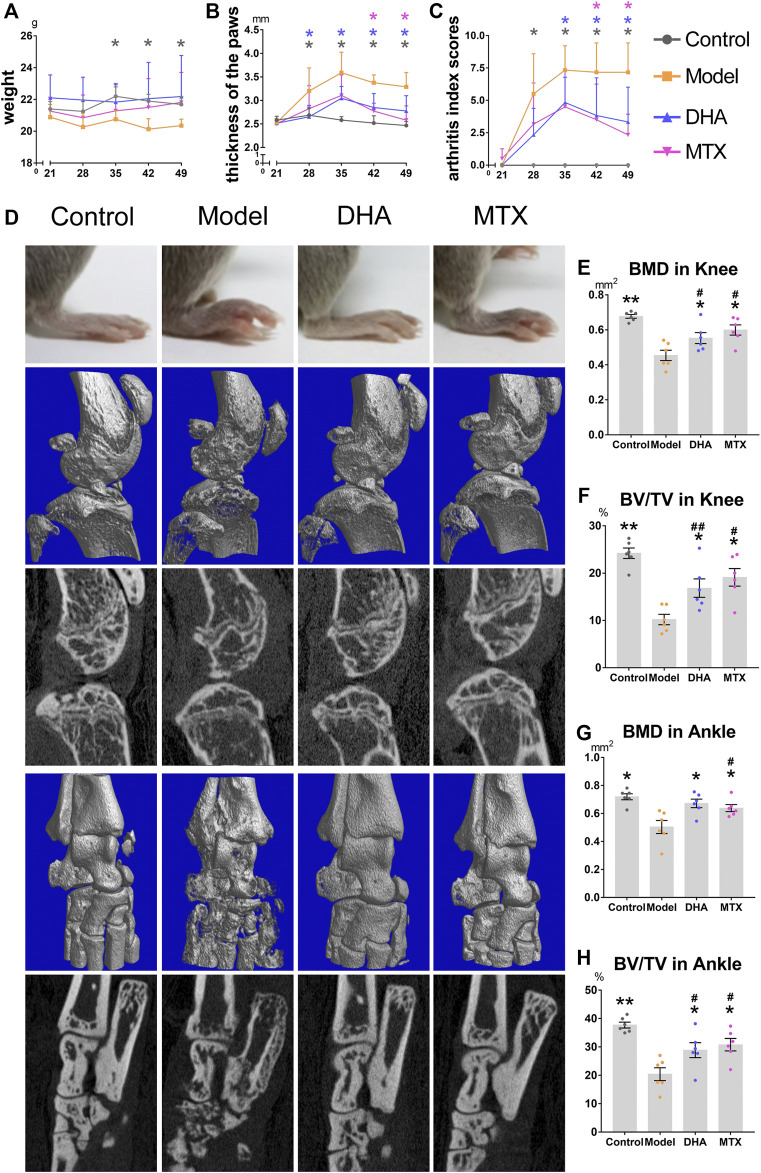
DHA alleviated inflammation and joint destruction in CIA mice. **(A)** Body weight were recorded weekly during the experiment. **(B)** The thickness of hind paws was measured weekly by digital calipers. **(C)** Arthritis index score was used to score each mice every week, with the highest score of 16. **(D)** Images of swollen hind paws of mice. The right hind ankle and knee of mice were scanned by the Skyscan 1176 Micro-CT Imaging System. **(E–H)** The BV/TV and BMD in knee and ankle were calculated. *p < 0.05, **p < 0.01 vs. the model group. ^#^ *p* < 0.05, ^##^ *p* < 0.01 vs. the control group.

DHA and MTX treatment did not cause weight loss in CIA mice (Figure 1A). Mice in DHA (20 mg/kg, daily), MTX (2 mg/kg, every 3 days) group showed significant reduction in paw oedema between day 28 and day 49 compared with mice in model group (*p* < 0.05, Figures 1B,C). Collagen challenge induced arthritis in mice, as evidenced by red swelling in the paw (Figure 1D). Micro-CT analysis showed that DHA or MTX treatment ameliorated the bone loss, as BMD and BV/TV in both DHA and MTX group were significantly higher than those in model group (*p* < 0.05, Figures 1D–H). Besides, no significant difference was observed between DHA and MTX groups in any of the above analyses.

### FcγRIIb mRNA Expression in Transfected ST486 Cells

**FIGURE 2:**
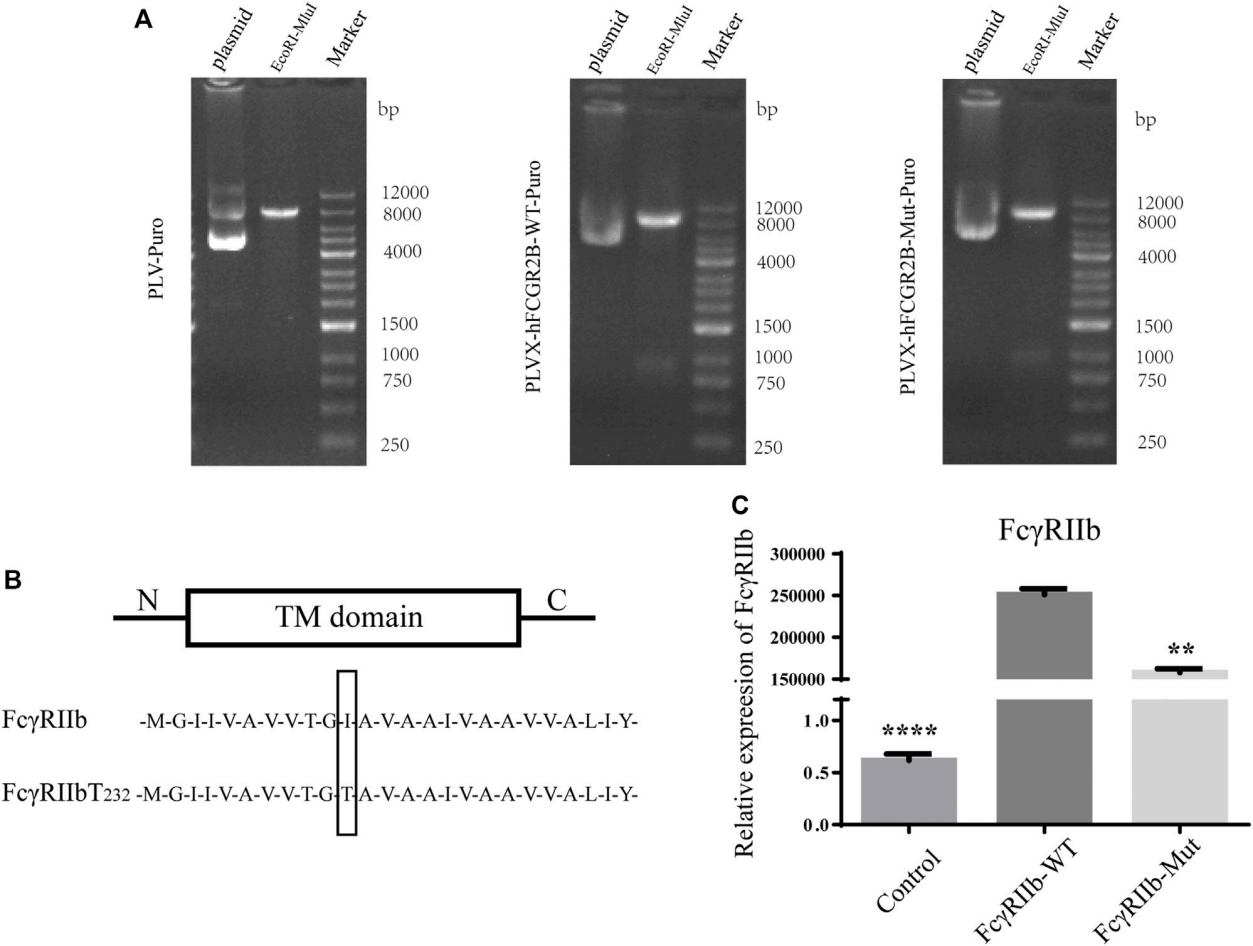
Design of the FcγRIIb-mutant cell lines. **(A)** Plasmid constructs: The human FcγRIIb coding gene was amplified by PCR and inserted into PLVX by EcoRI and MluI digestion sites. Obvious bands could be seen in 8000-bp and 1000-bp fragments, indicating that the 951-bp sequence of the target gene was successfully inserted. **(B)** FcγRIIb-Mut and FcγRIIb-WT encoded transmembrane proteins. Variants exist at locus 232. **(C)** Detection of plasmid transfection effect by qRT-PCR. The experiments were repeated three times and analyzed using one-way ANOVA. **p < 0.01, ****p < 0.0001 vs. the WT group.

As seen in Figure 2A, after the plasmid was digested, an obvious band could be seen around 8000bp, and there were 23bp bases between the two digestion sites, so it could not be detected. After the plasmids were digested by EcoRI + MluI, obvious bands could be seen at 8000 bp and 1000 bp attachment, indicating that the 951 bp sequence of the target gene had been inserted. The sequencing results showed correct and the plasmids were confirmed. Figure 2B shows the sequence maps of wild-type and variant FcγRIIb encoding membrane penetrating proteins, showing that variant FcγRIIb has a variation at site 232. As shown in Figure 2C, successful plasmid transfection was verified by qRT-PCR, and our results showed that when compared to the WT group, the relative expression of FcγRIIb was significantly decreased in both of the Con and Mut groups (*p* < 0.0001 and *p* < 0.01, respectively).

### Dihydroarteannuin Suppresses Proinflammatory Cytokine Expression Stimulated by Lipopolysaccharide

**FIGURE 3:**
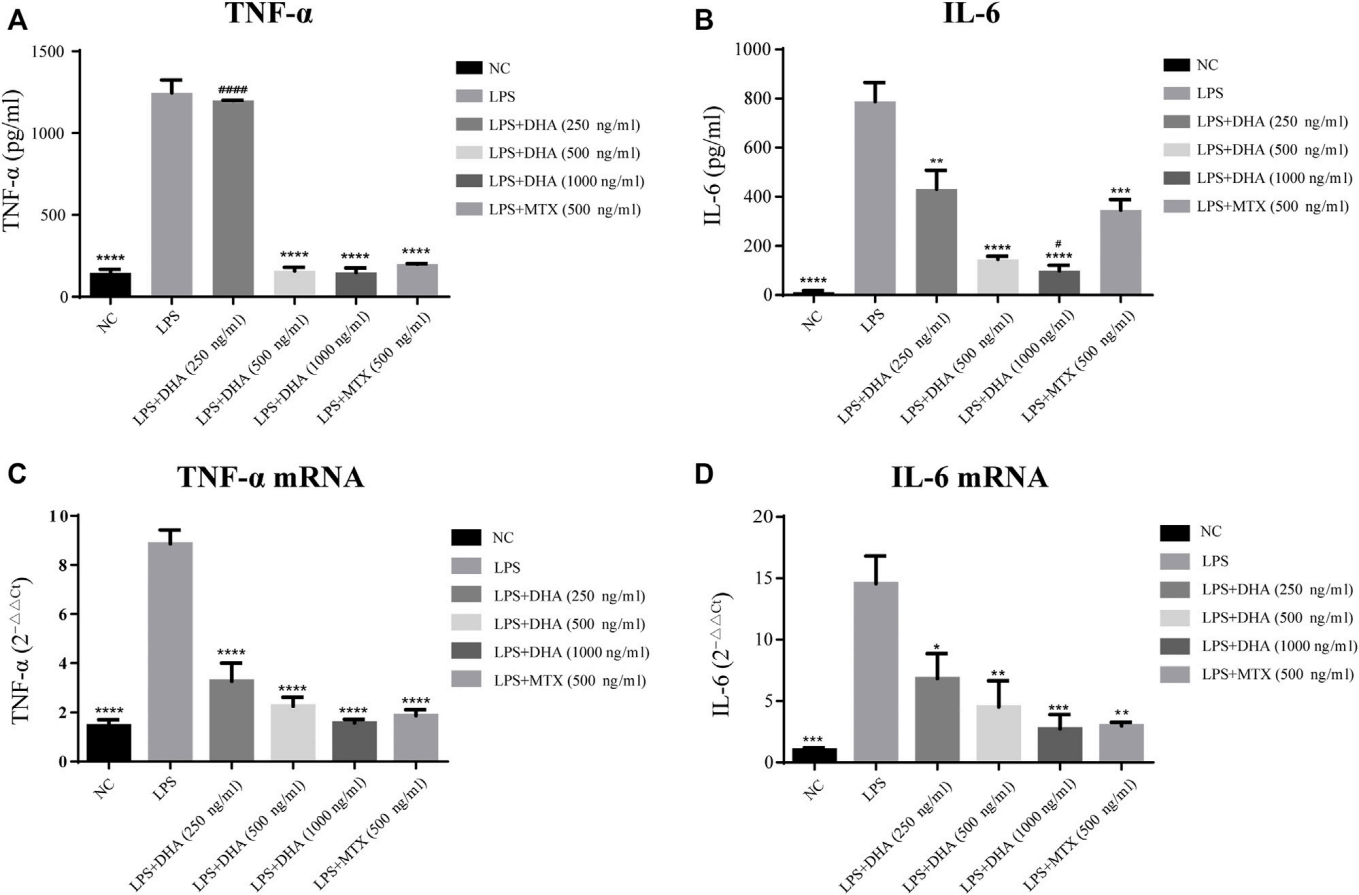
DHA inhibited the protein and mRNA expression of inflammatory cytokines in ST486. **(A,B)** The protein expression level of TNF-α and IL-6 were detected by ELISA. **(C,D)** The mRNA expression level of TNF-α and IL-6 were detected by qRT-PCR. The experiments were repeated three times and analyzed using one-way ANOVA. *p < 0.05, **p < 0.01, ***p < 0.001 and ****p < 0.0001 vs. the LPS group; ^#^ *p* < 0.05 and ^####^ *p* < 0.0001 vs. the MTX group.

Accumulated evidence reveals that certain proinflammatory cytokines contribute to the pathogenic factors supporting the proliferation and activation of B cells (Carvalho et al., 2011; Gottenberg et al., 2012; Lee et al., 2017). Therefore, we assessed the role of DHA in the regulation of inflammatory cytokine production by LPS-stimulated ST486 cells. As shown in Figures 3A,B, compared with the LPS group, the protein expression levels of TNF-α and IL-6 were significantly lower in both the DHA and MTX-treated groups, except for the protein expression level of TNF-α in the DHA (250 ng/ml) group. In addition, as seen in Figures 3C,D, we also found that IL-6 and TNF-α mRNA levels were decreased in the DHA-treated group compared to the LPS group, while the difference compared with the MTX group was not statistically significant.

### The Proliferation of ST486 Cells Were Suppressed Under Dihydroarteannuin Treatment

**FIGURE 4:**
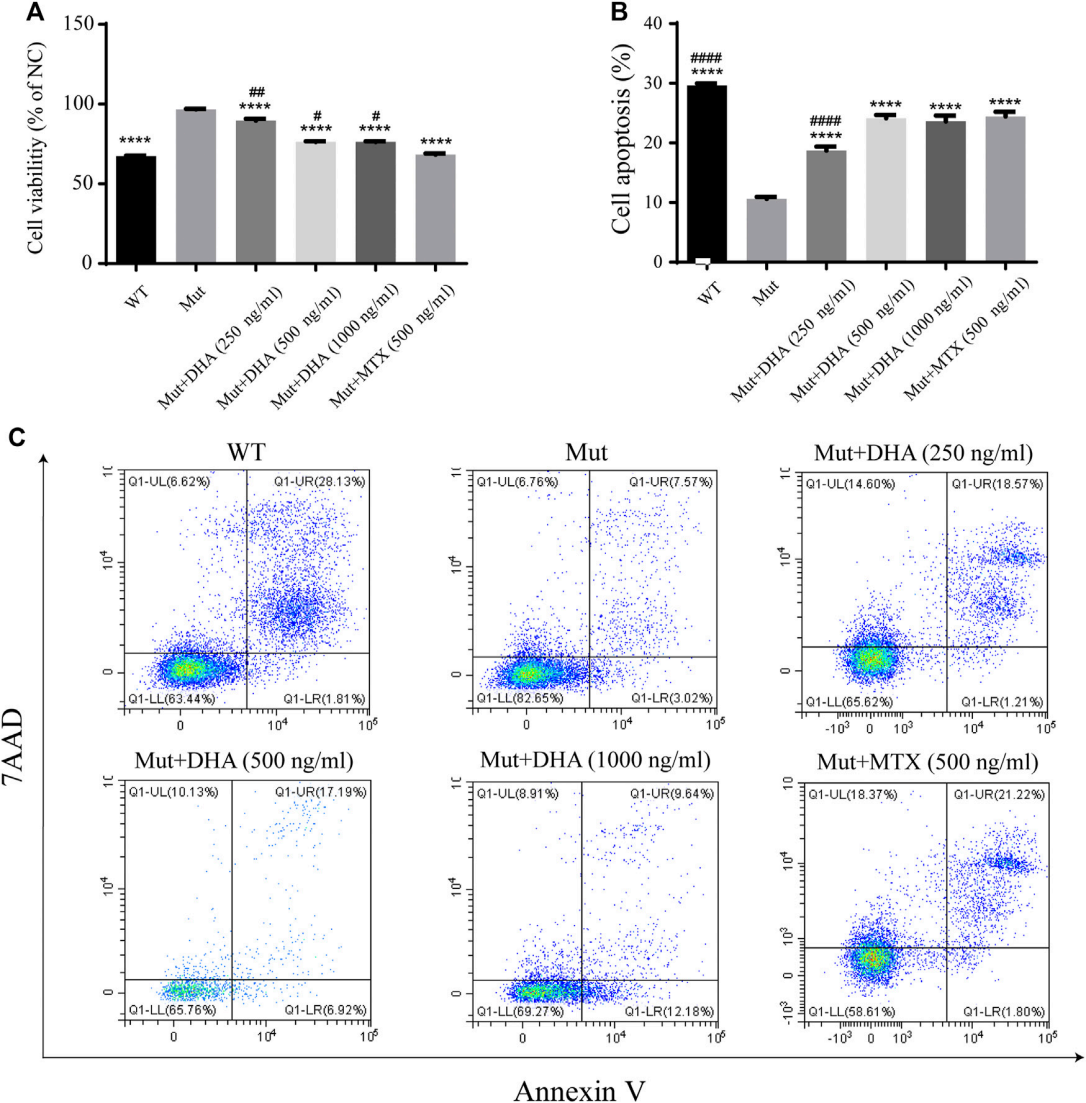
DHA inhibited the proliferation and promoted the apoptosis of ST486 cells. **(A)** The proliferation rate was determined by CCK-8 method. **(B,C)** Using Annexin V apoptosis detection kit, the apoptosis rate was detected according to the manufacturer’s instructions. The levels of Annexin V and 7AAD were detected by FACSCalibur flow cytometry. The experiments were repeated three times and analyzed using one-way ANOVA. ****p < 0.0001 vs. the Mut group. ^#^ *p* < 0.05, ^##^ *p* < 0.01 and ^####^ *p* < 0.0001 vs. the MTX group.

CCK-8 is used to detect the proliferation of ST486 cells and to evaluated the influence of DHA on the proliferation of ST486 cells. Figure 4A showed that when compared with those in the Mut group, the proliferation of ST486 in the WT, MTX and different dose of DHA goroup were all significantly decreased (*p* < 0.0001, *p* < 0.0001, *p* < 0.0001, *p* < 0.0001, and *p* < 0.0001, respectively). Besides, when compared with the MTX group, the proliferation of ST486 in the different dose of DHA group were all significantly increased (*p* < 0.01, *p* < 0.05, and *p* < 0.05, respectively).

### Dihydroarteannuin Promoted the Apoptosis of ST486 Cells

The apoptotic cells were detected by flow cytometry. As shown in Figures 4B,C, when compared with that in the Mut group, the number of apoptotic cells in the WT, MTX and DHA-treated groups were all significantly increased (*p* < 0.0001, *p* < 0.0001, *p* < 0.0001, *p* < 0.0001, and *p* < 0.0001, respectively). Additionally, when compared with the MTX group, the numbers of apoptotic cells in the DHA-treated (250 ng/ml) was significantly decreased (*p* < 0.0001), the numbers of apoptotic cells in the WT group was increased (*p* < 0.0001).

### Dihydroarteannuin Regulated the CD19 Pathway and Decreased Intracellular Ca^2+^ Flux in ST486 Cells

**FIGURE 5:**
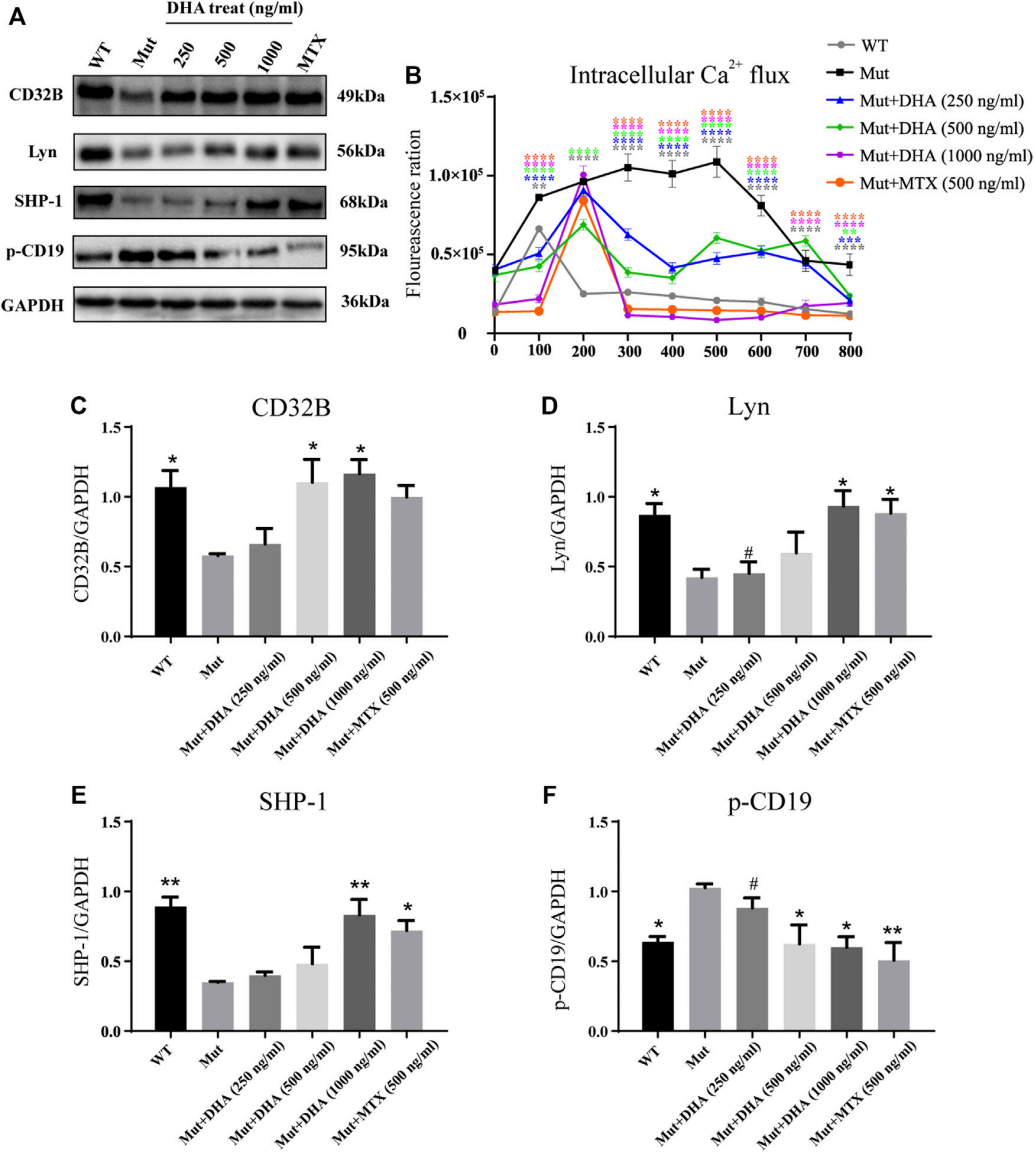
Regulation of DHA on CD19/FcγRIIb-Lyn-SHP-1 pathway and effect of intracellular Ca^2+^ flux in ST486 cells. **(A)** The cells were treated as above, and then stimulated with anti-human IgM (20 μg/ml) for 1 h. The protein expression levels of CD32b (FcγRIIb), GAPDH, Lyn, phospho-CD19 and SHP-1 were detected by western blot. This part of the experiment was repeated three times. **(B)** The cells were incubated in RPMI 1640 medium containing 10% fetal bovine serum and Fluo-4 AM (5 μmol), incubated at 37°C for 1 h, washed 3 times, and continuously monitored by flow cytometry. Anti-human IgM (20 μg/ml) was added at 50 s, add CaCl_2_ to the final concentration of 2 mM at 400 s, and the intracellular Ca^2+^ flux was continuously detected to 800 s **(C–F)** With GAPDH as the internal reference, the relative expression of each protein was expressed as the protein/GAPDH. The experiments were repeated three times and analyzed using one-way ANOVA. *p < 0.05 and **p < 0.01 vs. the Mut group; ^#^ *p* < 0.05 vs. the MTX group.

Previous studies have showed that FcγRIIb selectively dephosphorylated CD19 in consequence with an inhibition of BCR signaling (Koncz et al., 1998). Therefore, the western blot analysis was used to investigate whether the effect of DHA is correlated with the CD19 pathway. When compared to the cells in the WT group, the intracellular Ca^2+^ level in the Mut group was remarkably increased after 100 s. However, when compared with the Mut group, the calcium concentration decreased more rapidly in DHA and MTX group after 200 s (Figure 5B). Besides, as illustrated in Figure 5F, when compared with the Mut group, the protein expression levels of phosphorylated CD19 were all downregulated in the WT, DHA-treated (500 and 1000 ng/ml) and MTX groups (*p* < 0.05, *p* < 0.05, *p* < 0.05, and *p* < 0.01, respectively). These data all indicated that DHA inhibited B cell activation.

### Dihydroarteannuin Regulated the FcγRIIb/Lyn/SHP-1 Pathway in ST486 Cells

Previous studies have shown that FcγRIIb signal pathway plays an important role in B cell activation (Hippen et al., 1997), so we further study the effect of DHA on this signal pathway. (Figure 5A). The results showed that when compared to those in the Mut group, there were a significant increased in CD32B, Lyn and SHP-1 expression in the WT group (*p* < 0.05, *p* < 0.05, and *p* < 0.01, respectively, Figures 5C–E). Additionally, the protein expression of CD32B in DHA (500 and 1000 ng/ml) groups were much higher than that in the Mut group (*p* < 0.05 and *p* < 0.05, respectively). Besides, the Lyn expression in the DHA (1000 ng/ml) and MTX groups were significantly higher than that in the Mut group (*p* < 0.05 and *p* < 0.05, respectively). Correspondingly, when compared with the Mut group, consistent results for the SHP-1expression in the DHA (1000 ng/ml) and MTX groups (*p* < 0.01 and *p* < 0.05, respectively). In all, these data suggested that DHA impeded B cells activation induced by IgM through the modulation of the FcγRIIb/Lyn/SHP-1 pathway.

## Discussion

To date, DMARDs have been the primary choice for RA patient treatment, especially MTX, which is the anchoring drug of RA (Singh et al., 2015). However, MTX is a slow-acting anti-rheumatic drug, its effect is slow. In addition, MTX also has a large number of side effects. Therefore, it is urgent to develop alternative drugs with low toxicity. DHA is the most active component of artemisinin, it is extracted from traditional Chinese herbal medicine *Artemisia annua L*. Previous studies have shown that DHA has immunosuppressive effects on some diseases, such as RA and SLE. However, the exact molecular mechanism of DHA in treating RA is still unclear. In our study, we demonstrated that DHA achieved therapeutic effects in joint destruction in CIA mice. Besides, DHA could not only suppress the proinflammatory cytokine production of B cells but also promote the apoptosis, and inhibit the proliferation and activation of these cells. More importantly, we provided the first evidence that DHA might achieve these effects by activating the FcγRIIb/Lyn/SHP-1 pathway.

Strong evidence indicates that B cell activation play a key role in the occurrence, development and treatment of RA (van Gaalen et al., 2005; Nakou et al., 2009; Chatzidionysiou et al., 2016). Previous studies have focused on autoantigen-driven B cell activation, but recent studies have found that B cells from RA patients have defects in autoimmune tolerance during bone marrow development. During the development of B cells, pre-B cells in bone marrow can greatly reduce the production of autoreactive B cells through the mechanisms of receptor rearrangement, clone deletion and Anergy, which is a normal central immune tolerance. But in spite of this, a small number (6%) of autoreactive B cells were released to the periphery (von Boehmer and Melchers, 2010; Pillai et al., 2011). At this time, the mechanism of peripheral B cell immune tolerance plays a role, which is regulated by FcγRIIb. After BCR signal is activated by autoantigen, FcγRIIb inhibits CD19 phosphorylation by cross-linking with BCR, activates Lyn and immune receptor tyrosine inhibitory motif (ITIM), and recruits SHP-1. SHP-1 dephosphorylates multiple signal molecules, inhibits the metabolism of Ca^2+^, hinders the cascade of activation signals triggered by BCR, and induces immune tolerance (Karnell et al., 2014). However, the 695T > C (Ile232Thr) polymorphism in exon five of FcγRIIb gene could attenuate the signal of inhibitory receptor, aggravate the condition of RA and joint destruction. More importantly, a clinical study indicated that FcγRIIb can be regulated, showing dysfunction in active RA and normal function in inactive RA where the disease is controlled. Therefore, human Burkitt B lymphoma cells (lacking endogenous FcγRIIb gene) were transfected with a 232Thr loss-of-function mutant to construct a mutant cell model ST486 for cell experiments.

The level of inflammation plays an important role in the pathogenesis of RA and magnifying local tissue injury. In previous studies, found that DHA inhibited the release of TNF-α and IL-6 in a dose-dependent manner Li et al. (2008). DHA can also improve lupus symptoms in BXSB mice by inhibiting the production of TNF-α(Li et al., 2006). In the study, our results are similar to the findings of previous studies.

We further investigated the effects of DHA on the apoptosis and proliferation of ST486 cells. Our data show that DHA can not only inhibit the proliferation of ST486 cells, but also promote its apoptosis. We speculate that the effect of DHA on ST486 cells may be related to FcγRIIb/Lyn/SHP-1 pathway. Previous studies have shown that this signaling pathway is an important pathway for B cell activation, which is closely related to B cell activation and apoptosis (Xiang et al., 2007; Veri et al., 2010). Therefore, we further evaluated the effect of DHA on FcγRIIb/Lyn/SHP-1 signal pathway by WB, and found that the protein levels of CD32B(FcγRIIb), Lyn and SHP-1 were all up-regulated after DHA treatment. The results not only confirmed our hypothesis but were also consistent with previously published findings showing that FcγRIIb can be regulated (Magnusson et al., 2014).

Previous studies have shown that when cross-linked with BCR, FcγRIIb inhibits the activation of B cells by blocking the co-localization of CD19 and BCR (Xu et al., 2014). In order to further evaluate the specific mechanism of DHA in the treatment of RA, we studied the changes of CD19 phosphorylation level and intracellular Ca^2+^ after DHA intervention. Our data show that DHA can inhibit not only the phosphorylation level of CD19, but also the level of Ca^2+^. All these results indicate that DHA can inhibit the activation of B cells. Overall, our data demonstrate that DHA achieves these effects through a process that associated with the FcγRIIb/Lyn/SHP-1 pathway. However, there are some shortcomings in our study. This study did not involve the validation of *in vivo* molecular mechanisms, other than the effects of DHA on patients were not assessed in our study. Therefore, further clinical studies will be conducted to evaluate the clinical efficacy of DHA.

In conclusion, as far as we know, our study first demonstrated that DHA can not only reduce the level of inflammation in CIA mice, but also reduce the risk of bone destruction. In addition, we found that DHA can not only inhibit the production of B cell pro-inflammatory cytokines, but also inhibit B cell proliferation and activation, and promote B cell apoptosis. More importantly, we have demonstrated for the first time that DHA can inhibit B cell activation by activating FcγRIIb/Lyn/SHP-1 signaling pathway, thus achieving the therapeutic effect of RA. Therefore, our study highlights the potential of DHA as an alternative drug for RA.

## Data Availability

The original contributions presented in the study are included in the article/Supplementary Material, further inquiries can be directed to the corresponding author.

## Glossary

ACPA: anti-cyclic citrullinated peptide antibodies
BCR: B cell receptor
BMD: bone mineral density
BV/TV: bone volume/tissue volume
Bcl-2: B cell lymphoma-2
CCP: cyclic citrullinated peptide
CFA: complete Freund’s adjuvant
CIA: collagen-induced arthritis
DHA: dihydroarteannuin
DMARDs: disease-modifying antirheumatic drugs
ELISA: enzyme-linked immuno sorbent assay
FBS: fetal bovine serum
FcγRIIb: Fc gamma receptor b
IFA: incomplete Freund’s adjuvant
IL-6: interleukin-6
ITIM: immunoreceptor tyrosine-based inhibitory motif
LPS: lipopolysaccharide
Lyn: protein tyrosine kinases
LVX: lentiviral expression vector
MTX: methotrexate
MSCV: murine stem cell virus
PMA: phorbol myristate acetate
qRT-PCR: Quantitative real-time PCR
RA: rheumatoid arthritis
RF: rheumatoid factors
SHP-1: SH2-containing tyrosine phosphatase-1
SLE: systemic lupus erythematosus
SNP: single nucleotide polymorphism
SPF: specific pathogen-free
ST486: Burkitt’s lymphoma ST486 human B cells
TNF-α: tumor necrosis factor-alpha
WT: wild type
