# Supplementary material for: Dihydroarteannuin Ameliorates Collagen-Induced Arthritis Via Inhibiting B Cell Activation by Activating the FcγRIIb/Lyn/SHP-1 Pathway
Source: Front Pharmacol. 2022 May 3;13:883835. doi: 10.3389/fphar.2022.883835 (PMC9111742; doi:10.3389/fphar.2022.883835)
Supplement: Supplementary file 1 [file Table1.DOCX]

Supplementary Table 1

| Gene name | Primer sequence |
| --- | --- |
| FcγRIIb-WT | 5′-TGGTCACTGGGATTGCTGTA -3′(forward) |
|  | 5′-ATTGTGTTCTCAGCCCCAAC-3′(reverse) |
| FcγRIIb-Mut | 5′-ATGGGAATCCTGTCATTCTTACCTGT -3′(forward) |
|  | 5′-CTAAATACGGTTCTGGTCATCAGGC-3′(reverse) |
| beta-actin | 5′-ACTGGAACGGTGAAGGTGACA-3′ (forward) |
|  | 5′-TCGGCCACATTGCAGAACT-3′ (reverse) |
| TNF-α | 5'-CCTCTCTCTAATCAGCCCTCTG-3' (forward) |
|  | 5'-GAGGACCTGGGAGTAGATGAG-3' (reverse) |
| IL-6 | 5'-CCTGAACCTTCCAAAGATGGC-3' (forward) |
|  | 5'-TTCACCAGGCAAGTCTCCTCA-3' (reverse) |
| GAPDH | 5′-GGAGCGAGATCCCTCCAAAAT-3′ (forward) |
|  | 5′-GGCTGTTGTCATACTTCTCATGG-3′ (reverse) |

Primer sequences in this study.
